# Naturally Occurring Glycosidases in Milk from Native Cattle Breeds: Activity and Consequences on Free and Protein Bound-Glycans

**DOI:** 10.3390/metabo11100662

**Published:** 2021-09-28

**Authors:** Anne Vuholm Sunds, Ida Schwartz Roland, Ulrik Kræmer Sundekilde, Martin Nørmark Thesbjerg, Randall Robinson, Apichaya Bunyatratchata, Maria Glantz, Marie Paulsson, Daiva Leskauskaite, Anne Pihlanto, Ragnhild Inglingstad, Tove Gulbrandsen Devold, Gerd Elisabeth Vegarud, Bryndis Eva Birgisdottir, Maria Gudjonsdottir, Daniela Barile, Lotte Bach Larsen, Nina Aagaard Poulsen

**Affiliations:** 1 Department of Food Science, Aarhus University, Agro Food Park 48, 8200 Aarhus, Denmark; isr@food.au.dk (I.S.R.); uksundekilde@food.au.dk (U.K.S.); mnt@food.au.dk (M.N.T.); lbl@food.au.dk (L.B.L.); nina.poulsen@food.au.dk (N.A.P.); 2 Department of Food Science and Technology, University of California-Davis, Davis, CA 95616, USA; rcrobinson@ucdavis.edu (R.R.); dbarile@ucdavis.edu (D.B.); 3 Department of Food Technology and Nutrition, Faculty of Technology, Mahasarakham University, Maha Sarakham 44000, Thailand; abunyatratchata@ucdavis.edu; 4 Department of Food Technology, Engineering and Nutrition, Lund University, SE-22100 Lund, Sweden; maria.glantz@food.lth.se (M.G.); marie.paulsson@food.lth.se (M.P.); 5 Department of Food Science and Technology, Kaunas University of Technology, Radvilenu pl 19, LT-50254 Kaunas, Lithuania; daiva.leskauskaite@ktu.lt; 6 Natural Resources Institute Finland, Myllytie 1, FI-31600 Jokioinen, Finland; anne.pihlanto@luke.fi; 7 Faculty of Chemistry, Biotechnology and Food Science, Norwegian University of Life Sciences, NO-1432 Ås, Norway; ragnhild.aaboe@nmbu.no (R.I.); tove-gulbrandsen.devold@nmbu.no (T.G.D.); gerd.vegarud@nmbu.no (G.E.V.); 8 Faculty of Food Science and Nutrition, School of Health Sciences, University of Iceland, 113 Reykjavik, Iceland; beb@hi.is (B.E.B.); mariagu@hi.is (D.B.)

**Keywords:** natural glycosidases, oligosaccharides, *O*-linked glycans, principal component analysis, cattle breeds, mass spectrometry, NMR spectroscopy

## Abstract

Little is known about the extent of variation and activity of naturally occurring milk glycosidases and their potential to degrade milk glycans. A multi-omics approach was used to investigate the relationship between glycosidases and important bioactive compounds such as free oligosaccharides and *O*-linked glycans in bovine milk. Using 4-methylumbelliferone (4-MU) assays activities of eight indigenous glycosidases were determined, and by mass spectrometry and ^1^H NMR spectroscopy various substrates and metabolite products were quantified in a subset of milk samples from eight native North European cattle breeds. The results showed a clear variation in glycosidase activities among the native breeds. Interestingly, negative correlations between some glycosidases including β-galactosidase, *N*-acetyl-β-*d*-glucosaminidase, certain oligosaccharide isomers as well as *O*-linked glycans of κ-casein were revealed. Further, a positive correlation was found for free fucose content and α-fucosidase activity (r = 0.37, *p*-value < 0.001) indicating cleavage of fucosylated glycans in milk at room temperature. The results obtained suggest that milk glycosidases might partially degrade valuable glycans, which would result in lower recovery of glycans and thus represent a loss for the dairy ingredients industry if these activities are pronounced.

## 1. Introduction

Since domestication, cattle have been selectively bred to improve different production traits such as meat quality or milk yield [1]. In modern farming, cattle breeds that were originally native to specific farming regions have been replaced by high-yielding breeds, e.g., Holstein-Friesians, which are high in milk yield [1,2,3]. As a result, most of the native breeds have become endangered [2]. The breeds, however, present great phenotypic diversity reflected in different coat colours, sizes of horns and milk compositional traits. This diversity can be attributed to adaptations to distinct environments and management systems, genetic drift and isolation in local geographic areas. They are, therefore, a unique genetic resource due to their diverse gene pool and phenotypic characteristics [2,4]. 

Bovine milk oligosaccharides (OS) possess multiple bioactive effects. They have shown to modulate the gut microbiota [5], improve cognition and brain development [6,7] and positively affect intestinal health [8,9]. Bovine milk OS are thus of high interest as valuable dairy ingredients, with the potential to improve the composition and bioactive content of products such as infant formula. A previous study from this group comparing the OS among multiple breeds revealed that native breeds generally had a higher abundance of fucosylated OS in comparison to standard commercial bovine milk, and that the milk of Eastern Finncattle and Icelandic cattle was particularly high in acidic and fucosylated OS, respectively [10]. Knowledge of these differences in OS abundance among cattle breeds is valuable for acknowledgement of the breeds’ unique characteristics and possible exploitation for dairy products and ingredients. However, to understand the mechanisms affecting the observed variations, the activity of indigenous milk glycosidases must be taken into consideration. Indigenous glycosidases are naturally present in milk where they are expected to play a role in the digestion of sugars in the infant’s gastrointestinal tract [11], similar to the milk proteases which have been suggested to aid the release of functional peptides [12]. The glycosidases in milk can originate from the mammary cells, i.e., from epithelial or immune cells, and can further originate from the milk microbiota [13]. Enzyme activities may vary as a result of numerous factors, such as cow breed, lactation state, feeding system, season and health state of the cow [14,15,16,17]. The glycosidases *N*-acetyl-β-*d*-glucosaminidase and β-glucuronidase have been studied extensively, as these are effective markers of mastitis infections and are associated with high somatic cell count (SCC) [18,19].

Protein linked glycoconjugates are other valuable components in milk that possess similar functionalities to the free milk OS [20,21] and may be negatively affected by glycosidases. The *O*-linked glycans of ᴋ-casein (ᴋ-CN) were selected in this study to represent an example of a milk glycoprotein, which may be degraded by glycosidases. During cheese production, ᴋ-CN is cleaved by the enzyme chymosin at Phe^105^-Met^106^, resulting in the formation of *para*-κ-casein (Residues 1–105)—which will take part of cheese formation—and glycomacropeptide (GMP; Residues 106–169), which contains all the *O*-linked glycan residues, will remain soluble in the whey [22]. Due to the presence of various sialylated glycan structures, GMP has become a valuable dairy ingredient and is recovered from whey at a large scale [23]. ᴋ-CN has been found to have at least 11 genetic variants, denoted A, B, C, E, F^1^, F^2^, G^1^, G^2^, H, I, and J [24]. κ-CN is also a heterogeneous protein with several post-translational modifications, including 0–3 phosphorylations and 0–6 *O*-linked glycans. The five κ-CN glycosylation forms are referred to as the a, b, c, d and e types, constituting galactose (Gal), *N*-acetyl-galactosamine (GalNAc) and *N*-acetyl-neuraminic acid (sialic acid, NeuAc) [25,26,27], where a = GalNAc (Mw = 203.19 Da), b = Galβ(1–3)GalNAc (Mw = 365.34 Da), c = NeuAcα(2–3)Galβ(1–3)GalNAc (Mw = 656.59 Da), d = Galβ(1–3)[NeuAcα(2–6)]GalNAc (Mw = 656.59 Da), and e = NeuAcα(2–3)Galβ(1–3)[NeuAcα(2–6)]GalNAc (Mw = 947.85 Da). Further, an additional glycosylation form has been reported constituting *N*-acetyl-galactosamine and sialic acid (Mw = 494.45 Da) [28]. Six glycosylation sites on Variant B and seven on Variants A and E have been described [25,27]. ᴋ-CN glycosylation contains a high degree of sialic acid (attached to glycosylation sites c, d and e) [25]. It is well known that sialic acid-containing glycans provide significant health benefits, where a major focus has been on their role in brain development of neonates [29]. Free acidic OS extracted from bovine whey were shown to improve muscle, liver and brain metabolism in piglet models of infant undernutrition [7]. The present study investigated variations in glycosidase activities and naturally occurring OS, *O*-linked glycans of ᴋ-CN and monosaccharides, as well as the potential associations between glycan substrates (OS and *O*-linked glycans) and metabolite products, in milk from native cattle breeds, based on the same sample set as Sunds et al. [10].

## 2. Results 

### 2.1. Variation in Natural Milk Glycosidases among Native North European Cattle Breeds

**Figure 1:**
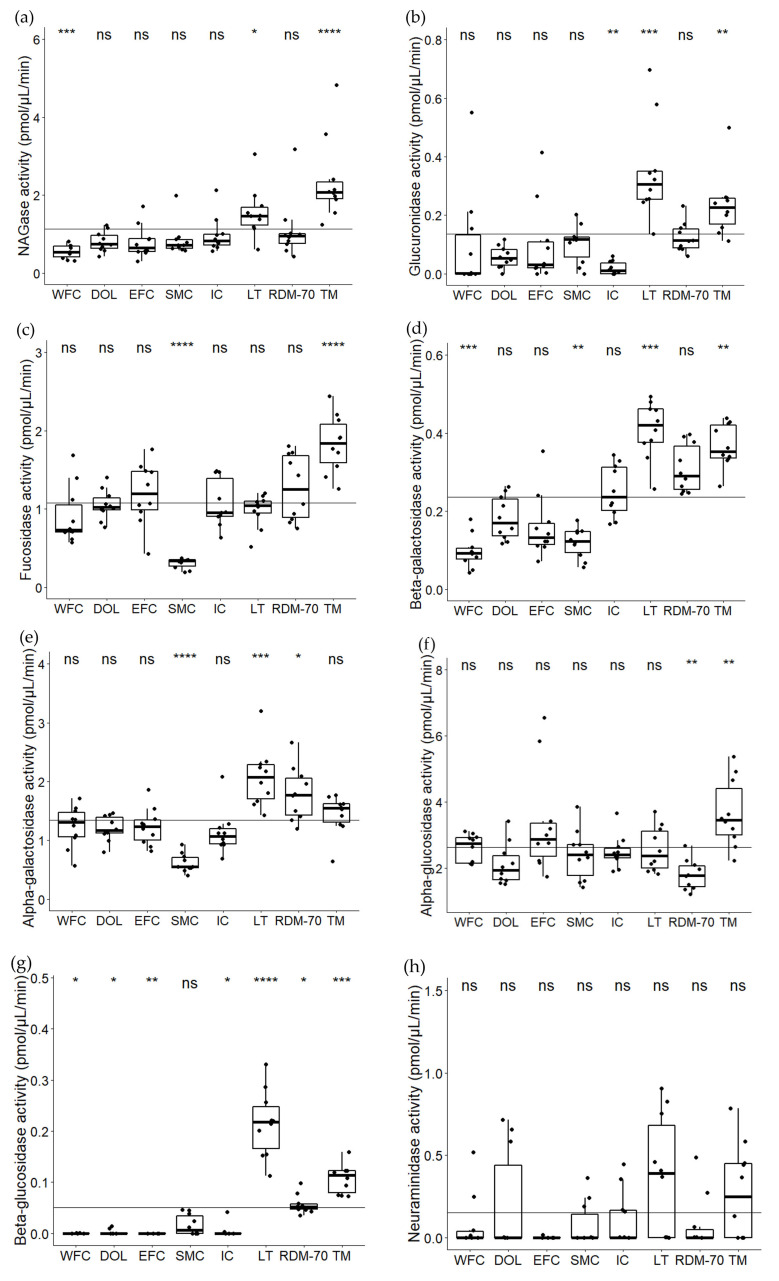
Glycosidase activities of (**a**) *N*-acetyl-β-glucosaminidase (NAGase), (**b**) β-glucuronidase, (**c**) α-fucosidase, (**d**) β-galactosidase, (**e**) α-galactosidase, (**f**) α-glucosidase, (**g**) β-glucosidase and (**h**) *N*-acetyl-α-neuraminidase, in milk from Western Finncattle (WFC), Norwegian Doela cattle (DOL), Eastern Finncattle (EFC), Swedish Mountain cattle (SMC), Icelandic cattle (IC), native Lithuania Black and White cattle (LT), Danish Red anno 1970 (RDM-70) and Norwegian Telemark cattle (TM). The horizontal line in each box indicates the median and the overall horizontal line indicates the mean of all samples in the study. Stars indicate significant differences from the overall mean: * *p* < 0.05, ** *p* < 0.01, *** *p* < 0.001, **** *p* < 0.0001, ns = not significant.

The variation in activities of eight glycosidases in milk from the eight native North European cattle breeds are shown in Figure 1. For all glycosidases studied, apart from *N*-acetyl-α-neuraminidase, at least one breed was found to be significantly different from the overall mean. The glycosidases varying the most across the studied breeds were α-fucosidase, β-galactosidase, *N*-acetyl-β-glucosaminidase and β-glucuronidase (CV% of 45%, 51%, 69% and 112%, respectively). Less variation was observed for α-glucosidase and α-galactosidase (CV% of 37% and 39% respectively). An intriguing finding was the high activities of most glycosidases in milk from Norwegian Telemark cattle, which displayed significantly higher activities compared to the overall mean for both *N*-acetyl-β-glucosaminidase, β-glucuronidase, α-fucosidase, β-galactosidase, α-glucosidase and β-glucosidase (*p*-values being <0.0001, 0.007, <0.0001, 0.002, 0.002 and 0.0008, respectively). Overall, high activities were observed in milk from native Lithuanian Black and White cattle, where significantly higher activities were found for *N*-acetyl-β-glucosaminidase, β-glucuronidase, β-galactosidase, α-galactosidase and β-glucosidase (*p*-values of 0.02, 0.0001, 0.0001, 0.0002 and < 0.0001, respectively), compared to the mean of all breeds. Finally, Danish Red anno 1970 milk displayed.

Significantly higher activities than the mean of all breeds for β-galactosidase, α-galactosidase and β-glucosidase (*p*-values of 0.05, 0.01 and 0.04, respectively). On the other hand, Swedish Mountain cattle milk revealed significantly lower activities compared to the mean of all breeds for α-fucosidase, β-galactosidase and α-galactosidase (*p*-values of <0.0001, 0.004 and <0.0001, respectively). Furthermore, low activities in Western Finncattle milk were measured for *N*-acetyl-β-glucosaminidase, β-galactosidase and β-glucosidase (*p*-values of 0.001, 0.0003 and 0.02, respectively). For the two glycosidases β-glucosidase and *N*-acetyl-α-neuraminidase no activities were detected in the majority of the samples; however minor activities were observed mainly in milk from Norwegian Telemark cattle and native Lithuanian Black and White cattle. These two cattle breeds were significantly more active in β-glucosidase compared to the overall mean. Almost no β-glucosidase activity was observed in milk from Western Finncattle, Norwegian Doela cattle, Eastern Finncattle and Icelandic cattle, which were all significantly lower than the overall mean (*p*-values of 0.004, 0.03, 0.004, 0.03, respectively), with only Swedish Mountain cattle being non-significant.

### 2.2. κ-Casein Isoform Variation across Native North European Cattle Breeds

The heterogeneous complexity of κ-CN among the native breeds was elucidated by LC-ESI/MS, revealing a total of 55 unique κ-CN isoforms across the dataset representing different combinations of genetic variants, glycosylations or phosphorylations. They contain from zero to three phosphorylations (0–3 P), and zero to three glycosylations. Due to similar masses of glycosylation (c) and glycosylation (d), it was not possible to distinguish between these glycans, and for the same reason it was not possible to distinguish between the di-glycosylated isoforms constituting either the two glycans (c/d, c/d) or (e, b). The following κ-CN genotypes were represented in the sample set: AA, AB, BB, AE and BE. Genotypes were identified based on molecular masses and retention times as compared to an in-house database, as previously conducted by Jensen et al. [26,30]. Variants A and B were identified in all breeds, whereas Variant E was only found in two Eastern Finncattle individuals with the AE and BE genotypes.

The majority of the GMP isoforms identified were phosphorylated. Based on the peak heights in MS chromatograms relative to the total peak height per sample it was revealed that the AA genotype expressed 99.2% phosphorylated isoforms of the total GMP across breeds, GMP AB expressed 96.7% and GMP BB expressed 93.2%. Single phosphorylations (1P) were most predominant representing 69–77% across breeds and genotypes. Double phosphorylations (2P) were the second most predominant isoforms representing 17–30%, and non-phosphorylated (0P) isoforms were represented only a small proportion: 0.8–6.8%. The tri-phosphorylated (3P) GMP isoforms were only found in the non-glycosylated forms and were in low prevalence. Across breeds, 40.7 ± 5.6% of Genotype AA were glycosylated on average, compared to 40.4 ± 8.0% of Genotype AB and 62.5 ± 13.4% of Genotype BB, thus in general GMP isoforms of genetic Variant B were more glycosylated compared to isoforms of genetic Variant A.

**Figure 2:**
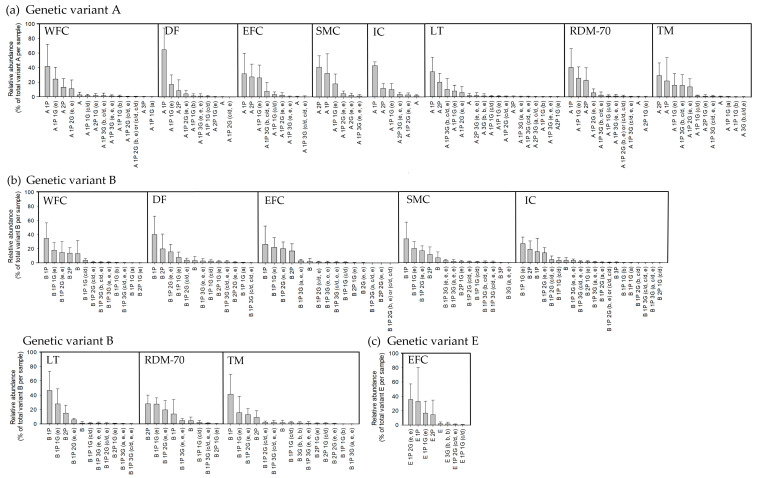
Prevalence of distinct glycomacropeptide (GMP) isoforms relative to total GMP (mean ± SD). (**a**) Genetic Variant A, (**b**) genetic Variant B and (**c**) genetic Variant E in milk from Western Finncattle (WFC), Norwegian Doela cattle (DOL), Eastern Finncattle (EFC), Swedish Mountain cattle (SMC), Icelandic cattle (IC), native Lithuania Black and White cattle (LT), Danish Red anno 1970 (RDM-70), Norwegian Telemark cattle (TM). A, B and E = genetic Variants A, B and E, P = phosphorylation, G = glycans, a–e = the five common *O*-linked glycans attached to κ-CN.

Figure 2 displays the distinct GMP isoforms identified for each breed within each genetic variant (A, B and E). Most isoforms were identified in genetic Variant B, where 9 to 20 isoforms were identified within each breed, compared to 6 to 18 for Variant A, and 8 for Variant E (only found in Eastern Finncattle milk). An exception was milk from the native Lithuanian Black and White cattle, which had seven extra compositions of glyco-phospho-forms in Variant A (18 isoforms) compared to Variant B (11 isoforms). Further, one and two more isoforms were found in Western Finncattle milk and Danish Red anno 1970 milk of Variant A compared to Variant B, respectively. The non-glycosylated GMP 1P was the most predominant isoform in most breeds, followed by either GMP 2P or GMP 1P (e). The GMP 2P was most prevalent in three breeds: Swedish Mountain cattle for Variant A, Norwegian Telemark cattle for Variant A, and Danish Red anno 1970 for Variant B. In total, three glycosylated isoforms were found with 0P, 12 glycosylated isoforms with 1P and three with 2P for genetic Variant A among all breeds. For genetic Variant B, three glycosylated isoforms were found with 0P, 15 with 1P and three with 2P, among all breeds. Among the glycosylated isoforms the most prevalent one observed across the breeds were GMP 1P (e) and GMP 1P (e, e), and these were mainly followed by the isoforms GMP 1P (c/d), GMP 1P (e, e, e) and GMP 1P (b, c/d, e). Notably, the triple-glycosylated isoform GMP 1P (b, c/d, e), which was relatively more prevalent for Variant A, was identified in 5 breeds and among the three most prevalent glycosylated isoforms for Variant A in these breeds. This specific glycosylated isoform was particularly prevalent in milk from native Lithuanian Black and White and Norwegian Telemark cattle, constituting 10% and 15.5% of the total GMP isoforms in these breeds, respectively. For Variant B, the isoform GMP 1P (b, c/d, e) was identified in only native Lithuanian Black and White and Norwegian Telemark cattle and was the fifth and eighth most abundant glycosylated isoforms in milk from these two breeds. Overall, three glycosylation compositions were solely found in milk of genetic Variant B; these isoforms were B 1P (a, e), B 1P (b, c/d) and B 1P (a, c/d, e). The primary glycosylated isoforms identified in Icelandic cattle milk were highly sialylated: GMP 1P (e), GMP 1P (e, e), GMP 1P (c/d, e) and GMP 1P (c/d, c/d), with the most abundant isoform being GMP 1P (e). No significant differences in the overall degree of κ-CN glycosylation, as determined from GMP, were observed among breeds.

### 2.3. Lactose and Monosaccharides in Milk from the Native North European Cattle Breeds

**Figure 3:**
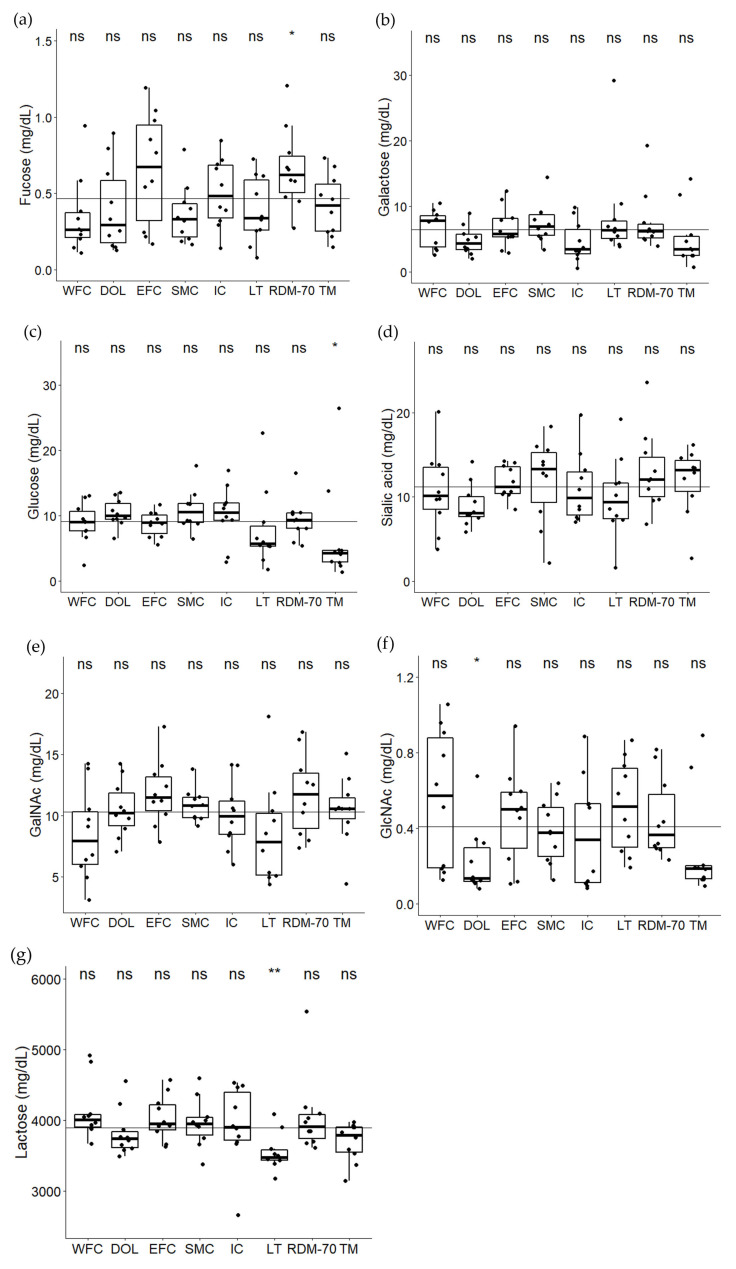
Free sugars analyzed by ^1^H NMR spectroscopy. (**a**) fucose, (**b**) galactose, (**c**) glucose, (**d**) sialic acid, (**e**) *N*-acetyl-galactosamine (GalNAc), (**f**) *N*-acetyl-glucosamine (GlcNAc) and (**g**) lactose, in milk from Western Finncattle (WFC), Norwegian Doela cattle (DOL), Eastern Finncattle (EFC), Swedish Mountain cattle (SMC), Icelandic cattle (IC), native Lithuania Black and White cattle (LT), Danish Red anno 1970 (RDM-70) and Norwegian Telemark cattle (TM). The horizontal line in each box indicate the median and the overall horizontal line indicates the mean of all samples in the study. Stars indicate significant differences from the overall mean: * *p* <0.05, ** *p* < 0.01, ns = not significant.

By use of ^1^H NMR spectroscopy, lactose and monosaccharides (fucose, galactose, glucose, *N*-acetyl-glucosamine (GlcNAc), *N*-acetyl-galactosamine (GalNAc), *N*-acetyl-neuraminic acid, (NeuAc)) were identified and quantified (Figure 3). For galactose, *N*-acetyl-galactosamine, and sialic acid, no breed had concentrations significantly different from the overall mean. Fucose was significantly more abundant in Danish Red anno 1970 milk (*p* = 0.03) compared to the overall mean. Glucose was significantly lower than the overall mean in Norwegian Telemark milk (*p* = 0.02), and lactose was significantly lower than the overall mean in native Lithuanian Black and White milk (*p* = 0.004). However, for all monosaccharides detected the intra-group variance was found to be relatively high, and one should be careful by making strong conclusions based on these results. 

### 2.4. Correlations between Milk Glycosidases and Glycans

**Figure 4:**
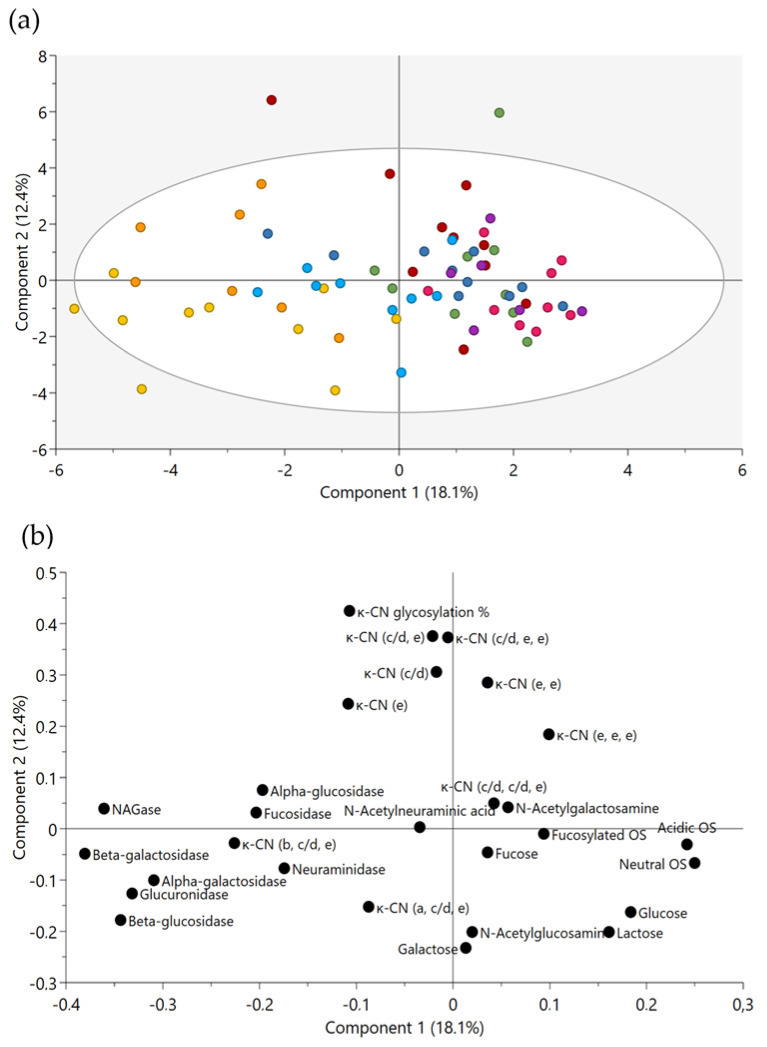
Principal component analysis (PCA) of the first two principal components. The PCA model includes relative abundances of neutral, acidic and fucosylated oligosaccharides (OS), nine GMP glycoforms ((e), (c/d), (e, e), (c/d, e), (c/d, e, e), (e, e, e), (b, c/d, e), (a, c/d, e) (c/d, c/d, e)), GMP glycosylation degree (%), six monosaccharides (fucose, galactose, glucose, *N*-acetyl-galactosamine (GalNAc), *N*-acetyl-glucosamine (GlcNAc), sialic acid), lactose and eight glycosidases (*N*-acetyl-β-glucosaminidase, β-glucuronidase, α-fucosidase, β-galactosidase, α-galactosidase, α-glucosidase, β-glucosidase, *N*-acetyl-α-neuraminidase). (**a**) Scores plot illustrating individual cows from each breed; Western Finncattle (WFC, pink), Norwegian Doela cattle (DOL, green), Eastern Finncattle (EFC, dark blue), Swedish Mountain cattle (SMC, purple), Icelandic cattle (IC, red), native Lithuania Black and White cattle (LT, yellow), Danish Red anno 1970 (RDM-70, light blue) and Norwegian Telemark cattle (TM, orange) (*n* = 70) and (**b**) corresponding loading plot.

A two-component PCA model was constructed, based on the quantitative data of glycosidases and glycans including; OS, *O*-linked glycans attached to GMP isoforms, monosaccharides and lactose. The model explains 18.1% and 12.4% of the total variation on the two first principal components, respectively (PC1 and PC2; Figure 4a,b).

The model revealed no clustering of the breeds, and thus no significant differences among groups. However, some breeds revealed a relatively low within-breed variation. The corresponding loadings were overall positioned as follows: OS in the positive end of PC1, GMP in the positive end of PC and glycosidase activities in the negative end of PC1.

Three breeds, native Lithuanian Black and White cattle, Norwegian Telemark cattle and Danish Red anno 1970 were more closely related to the loadings of the eight glycosidases positioned in the negative area of PC1, and at the same time affected by the low content of OS and free monosaccharides. Further, a few individuals from the two breeds Icelandic cattle and Norwegian Doela cattle were located towards the positive area of PC2, which is connected to the loading of the GMP glycosylation degree and the majority of the GMP glycosylated isoforms. Sialic acid and fucose did not contribute to the variation described by the model.

Across the data set, several significant correlations were observed. Among the significant correlations found, the 11 correlations considered to be of major biological relevance were selected and evaluated based on Pearson’s correlation coefficients, as reported in Table 1. Four relations between different glycosidase activities revealed moderately strong (r = 0.5–0.8) correlations; β-galactosidase activity correlated with β-glucosidase activity (r = 0.79, *p* < 0.001), β-glucuronidase activity with β-glucosidase activity (r = 0.73, *p* < 0.001), β-glucuronidase activity with β-galactosidase activity (r = 0.61, *p* < 0.001) and β-glucuronidase activity correlated with *N*-acetyl-β-glucosaminidase activity (r =0.52, *p* < 0.001).

**Figure 5:**
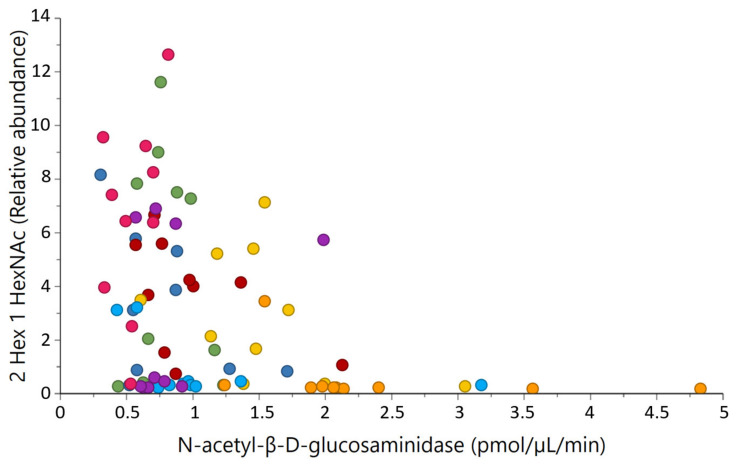
Relationship between the relative abundance of the neutral oligosaccharide 2 Hex 1 HexNAc and *N*-acetyl-β-glucosaminidase activity (r = −0.40, *p*-value < 0.001). Each native breed is represented by a colour; Western Finncattle (WFC, pink), Norwegian Doela cattle (DOL, green), Eastern Finncattle (EFC, dark blue), Swedish Mountain cattle (SMC, purple), Icelandic cattle (IC, red), native Lithuania Black and White cattle (LT, yellow), Danish Red anno 1970 (RDM-70, light blue) and Norwegian Telemark cattle (TM, orange).

**Figure 6:**
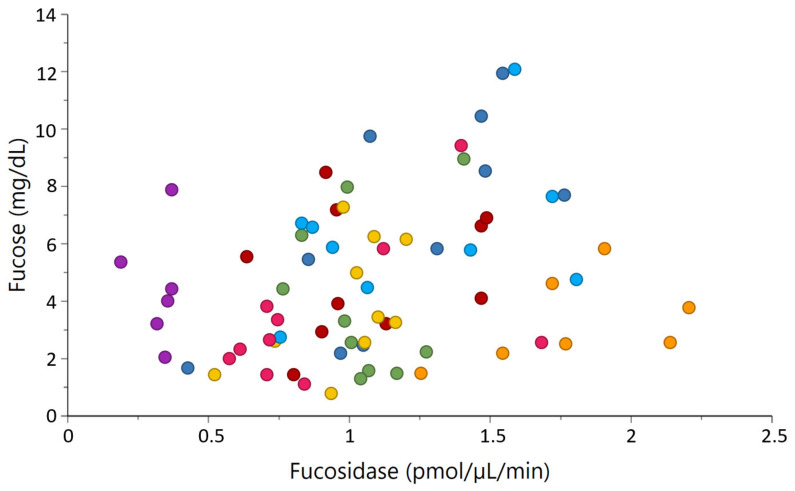
Relationship between the concentration of fucose and α-fucosidase activity (r = 0.37, *p*-value < 0.001). Each native breed is represented by a colour; Western Finncattle (WFC, pink), Norwegian Doela cattle (DOL, green), Eastern Finncattle (EFC, dark blue), Swedish Mountain cattle (SMC, purple), Icelandic cattle (IC, red), native Lithuania Black and White cattle (LT, yellow), Danish Red anno 1970 (RDM-70, light blue) and Norwegian Telemark cattle (TM, orange).

**Figure A1:**
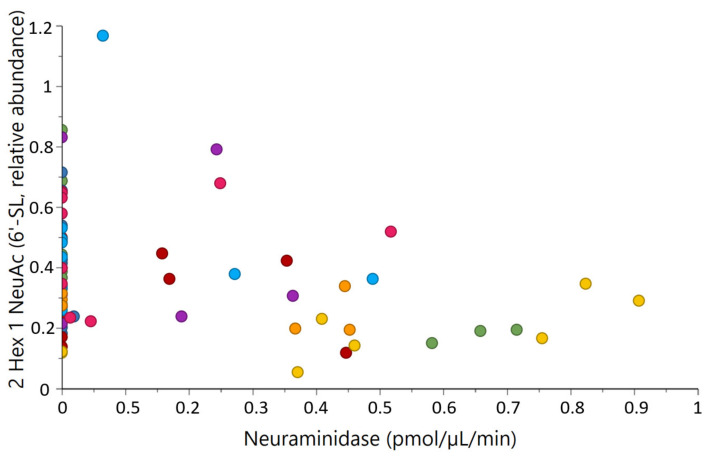
Relationship between the relative abundance of the acidic oligosaccharide 2 Hex 1 NeuAc (6-SL) and *N*-acetyl-α-neuraminidase activity (r = −0.27, *p*-value =0.024). Each native breed is represented by a colour; Western Finncattle (WFC, light red), Norwegian Doela cattle (DOL, green), Eastern Finncattle (EFC, dark blue), Swedish Mountain cattle (SMC, purple), Icelandic cattle (IC, red), native Lithuania Black and White cattle (LT, yellow), Danish Red anno 1970 (RDM-70, light blue) and Norwegian Telemark cattle (TM, orange).

Three out of the 11 major correlations between glycosidases and glycans were selected and presented in Figure 5 and Figure 6, as well as in Appendix A Figure A1, as these relations were of high biological interest. Figure 5 shows the major neutral OS with composition 2 Hex 1 HexNAc, which was negatively correlated with *N*-acetyl-β-*d*-glucosaminidase activities (r = −0.40, *p* < 0.001), indicating a cleavage of HexNAc residues. Figure A1 displays the negative correlation between one of the major the acidic OS, 6′-sialyllactose (6′-SL), and activities of *N*-acetyl-α-neuraminidase (r = −0.27, *p* = 0.024).

Figure 6 shows the positive correlation observed between α-fucosidase activity and its product free fucose (r = 0.37, *p* < 0.001). However, no clear correlation was observed between α-fucosidase activity and fucosylated OS. The strongest correlation between GMP isoforms and glycosidases was observed for GMP (e, e, e), which negatively correlated with β-galactosidase activity (r = −0.25, *p* = 0.030). Finally, the strongest correlations among glycans were observed for 2 Hex 2 NeuAc and GMP (c/d) (r = 0.36, *p* = 0.001), as well as for 3 Hex 1 NeuAc and GMP (c/d) (r = 0.33, *p* = 0.001).

## 3. Discussion

### 3.1. Variations in Milk Glycosidase Activities among Native North European Cattle Breeds

The expression of glycosidases in somatic cells from bovine milk has been documented by Wickramasinghe et al. [31]. Further, the presence of glycosidase activities in bovine and human milk has previously been demonstrated by O’Riordan et al. [11] and, Wiederschain and Newburg [32], respectively. In those studies, activities of α-fucosidase, α-galactosidase, β-galactosidase, β-glucosidase, *N*-acetyl-β-glucosaminidase, β-glucuronidase and *N*-acetyl-α-neuraminidase were monitored, with the activities of α-fucosidase and *N*-acetyl-α-neuraminidase being highest in mature bovine milk, and α-fucosidase and *N*-acetyl-β-glucosaminidase being highest in mature human milk [11,32]. More or less in line with those findings, the most active glycosidases in the milk from native cattle breeds were; *N*-acetyl-β-glucosaminidase, α-glucosidase, α-fucosidase and α-galactosidase, whereas overall low activities were obtained for β-glucosidase and *N*-acetyl-α-neuraminidase. Milk glycosidases are, in general, low in activity, as compared to, for example, milk proteases [12]. However, we still consider these to be of importance due to the intrinsic low concentration of their substrates, such as OS and glycoconjugates, which are valuable milk ingredients for the dairy industry.

The two glycosidases *N*-acetyl-β-*d*-glucosaminidase and β-glucuronidase showed the greatest variation in activity across breeds (CV% of 69% and 112%) and revealed significantly higher activities in milk from native Lithuanian Black and White cattle and Norwegian Telemark cattle, compared to the overall mean. These two enzymes are well-known indicators of mastitis and are known to be correlated with SCC in studies on mastitic milk [18,33,34]. While *N*-acetyl-β-*d*-glucosaminidase is known to able to cleave both terminal *N*-acetyl-galactosamine and *N*-acetyl-glucosamine from for example OS due to its unspecific behavior [35], β-glucuronidase is not relevant for OS degradation as the OS do not contain β-glucuronic acid. However, β-glucuronidase may cleave other milk glycans, such as the bovine milk glycosaminoglycan, hyaluronan, constituting dimers of β-glucuronic acid and *N*-acetyl-glucosamine [36].

*N*-acetyl-β-glucosaminidase activities in mastitic milk vary substantially among studies, and levels between 2–35 pmol/µL/min have been reported [18,37]. *N*-acetyl-β-glucosaminidase activities measured in this study vary between 0.3–4.8 pmol/µL/min. Nine samples were above 2 pmol/µL/min and may reflect a mild udder infection. Most of these milk samples belonged to the Norwegian Telemark cattle. Indicating that some individuals have a mild infection, which may be linked to the milking procedure since this breed was the only one milked by hand and not automatic. Furthermore, this breed had been grazing 4–5 days prior to sampling, which may also affect the enzymatic composition. The rest of the samples were within the normal range for low SCC milk. β-glucuronidase activities were in the range of 0–0.69 pmol/µL/min. In a previous study by Sunds et al. [37], levels of β-glucuronidase in subclinical mastitic milk were above 0.2 pmol/µL/min and in clinical mastitic milk the range was 1–1.2 pmol/µL/min. Twenty-two samples had β-glucuronidase activities above 0.2 pmol/µL/min, hence these samples may reflect cows with a subclinical mastitis infection, mainly belonging to native Lithuanian Black and White cattle and Norwegian Telemark cattle. Levels of SCC were unfortunately not monitored in this study. The strong positive correlations observed among several glycosidases of the study reflect that the mechanisms initiating their expression or leakage into the milk are similarly determined.

### 3.2. Post-Translational Modifications of κ-Casein among Native Breeds

Since no correlations were revealed between fucosylated OS and α-fucosidase activity, but significant correlations were revealed for both sialylated OS and neutral OS with the glycosidases cleaving sialic acid and *N*-acetyl-hexosamine, respectively, *O*-linked glycans were chosen for the study due to their high content of sialic acids. κ-CN was selected as a representative of glycoproteins decorated by *O*-linked glycans. Because κ-CN heterogeneity is well known, we also focused on elucidating variations in κ-CN and its derived GMP isoforms among native North European cattle breeds. Among the native breeds, between 93% and 99% of the total pool of κ-CN was phosphorylated, which is in agreement with levels reported in Danish Holstein milk by Jensen et al. [30], where 95–96% of κ-CN was phosphorylated. In the present study, the proportion of unmodified ᴋ-CN was lowest in genetic Variant A (0.8 ± 1.1%) and highest in genetic Variant B (6.8 ± 4.5%). A similar distribution was observed in Danish Jersey milk assessed by Jensen et al. [30], where ~10% of genetic Variant A was unmodified in Jersey milk and ~3% of Variant B was unmodified in Danish Holstein milk. Further, the present study identified four breeds that expressed tri-phosphorylated ᴋ-CN. A limited number of tri-phosphorylated ᴋ-CN isoforms have previously been identified in samples from Danish Holstein [26] and Swedish Red [38].

The average degree of κ-CN glycosylation among breeds was 46 ± 12%, with highest glycosylation of genetic Variant B. Jensen et al. [30] reported levels of 34–36% including all genetic variants in Danish Jersey and Danish Holstein milk based on 2D gels, and slightly more glycosylation could also be ascribed to genetic Variant B. The most dominating glycosylated isoforms ᴋ-CN 1P (e) and ᴋ-CN 1P (e, e), which have been confirmed by other studies on milk from Swedish Red, Holstein, Normande and Montbéliarde cattle [38,39]. The triple glycosylated isoform ᴋ-CN 1P (b, c/d, e) was highly prevalent in genetic Variant A compared to Variant B for the breeds Western Finncattle, Eastern Finncattle, native Lithuanian Black and White, Danish Red anno 1970 and Norwegian Telemark cattle. Despite its relatively high prevalence in the present study, this combination of glycans was not identified by several other studies [26,40,41].

Icelandic cattle milk contained the highest number of κ-CN isoforms, and further contained a high prevalence of sialylated species. These findings may reflect that the majority of the cows within this breed were of Genotype BB (*n* = 8), which were generally more glycosylated. However, it may also be a result of other biological factors, such as breed-specific differences, as Iceland cattle milk has also been found to be higher in abundance of a free fucosylated OS (3 Hex 6 HexNAc 1 Fuc, *p* = 0.037) [10]. This may indicate that Icelandic cattle in general possess glycans with a high complexity and with a relatively high proportion of functional monosaccharide residues, such as sialic acid and fucose. For ᴋ-CN genetic Variant E, the most dominating isoform possessed double glycosylations, containing four sialic acid residues in total, GMP 1P (e, e), whereas for Variants A and B, the most dominating isoform displayed singly glycosylated GMP 1P (e), containing two sialic acids. The same overall distribution of sialic acid-containing GMP among the genetic variants was observed in an industrial GMP isolate by Sunds et al. [42]. Mainly the GMP isoforms containing either glycan c or d correlated positively with the OS. Strongest were the correlations to the isomers 2 Hex 2 NeuAc and 3 Hex 1 NeuAc, suggesting that the specific enzymes involved in the synthesis of these glycans are in common.

### 3.3. Potential Associations of Natural Milk Glycosidases and Glycans

The two-component PCA model revealed interesting correlations among the loadings, which report the values for glycosidase activities, OS, metabolite products and GMP profiles, indicating that these variables vary in the milk samples. Overall, milk with high glycosidase activity were characterized by low content of OS, since these loadings were positioned in opposite corners of the plot, as described by PC1. A relatively low level of explained variance is expected with the large number of variables included, and it also indicates that other trends remain in the data. Western Finncattle and Swedish Mountain cattle were mainly positioned in the positive area of PC1. However, the position of Western Finncattle milk samples indicates that this breed expresses low levels of glycosidases and that the glycosidases found to be active in the milk have not degraded the OS, which were present in a high abundance. Swedish Mountain cattle milk, on the other hand, was relatively low in abundance of OS, and its position in the PCA plot reflect its low activities in several glycosidases compared to the other breeds. This observation is opposite to particularly the breeds Norwegian Telemark cattle and native Lithuanian Black and White cattle, which were associated with high glycosidase activities. Interestingly, the triple-glycosylated isoform GMP 1P (b, c/d, e), which was prevalent relative to several other glycosylated isoforms in Variant A milk, was particularly prevalent in native Lithuanian Black and White cattle and Norwegian Telemark cattle milk. Similar to most other *O*-linked glycosylations on GMP, the GMP 1P (b, c/d, e) did not seem to be affected by the glycosidases, which may illustrate that these isoforms are relatively resistant to hydrolysis compared to some of the OS. For future studies it is of relevance to include *N*-glycans as well. The PCA plot indicated a negative relationship between neutral OS and most of the glycosidases, and similar for acidic OS and most glycosidases. The correlations are, however, not strong and it should be emphasized that the sample set includes several inherent biological variations, such as breed differences, broad lactation state range, parity range, feed and age, which could influence these relations. Hence to obtain more solid conclusions a larger and more balanced data set is needed. Such an experiment is, however, challenging when studying endangered native breeds, where the number of animals is critically low and all are not necessarily milked. However, in the future they may be bred for unique beneficial traits, such as high milk glycan levels, to serve as sources for valuable dairy ingredients.

Lactation stage affects the concentration of OS negatively in Holstein and Jersey milk, opposite to the gene expression of glycosidases which increase over lactation [31]. These trends may also explain the negative relations observed for some OS and glycosidases in the present study. However, in the present study no significant correlations were revealed between lactation stage and glycosidase activities, nor between lactation stage and OS abundance. Decreasing activities over lactation (0 to 90 days) were reported by O’Riordan et al. [11] for most glycosidases. This is opposite to the increasing expression of glycosidases over lactation, indicating that they may be increasingly expressed in milk but not equivalently activated. The negative relations of certain OS and glycosidases observed are hence more likely to result from degradation than from reverse expression patterns. Glycosidases have been postulated to be expressed in milk to help the neonate degrade the indigestible OS until a healthy gut microbiota is established, which in calves takes approximately seven days post-parturition [11,43]. Thus, from a biological point of view the glycosidases are only needed in early life. Another role of the glycosidases is to remodel larger glycan structures synthesized in the Golgi apparatus and trimmed by glycosidases in the lysosomes in formation of smaller *N*-glycans [44]. Besides lactation stage, season and feed have also shown to affect glycosidase activities and may thus be a part of the explanation for these trends in the data [16,17].

No significant variations in levels of sialic acid were revealed among the native breeds and *N*-acetyl-α-neuraminidase was either not active or active at low levels. This is in contrast to the study by O’Riordan et al. [11] where relatively high activities of *N*-acetyl-α-neuraminidase were revealed in bovine milk, reported to be the second most active in mature milk (Day 90). The high prevalence of acidic OS in bovine milk, as compared to human milk, may be explained by the low activity levels of *N*-acetyl-α-neuraminidase observed in the present study, which are thus not present to cleave terminal sialic acid residues from the bovine milk OS in the same degree as in human milk. A negative correlation was observed between 6′-SL and *N*-acetyl-α-neuraminidase, whereas the major acidic OS in bovine milk, 3′-SL, did not significantly correlate with *N*-acetyl-α-neuraminidase. This suggests that the milk neuraminidases monitored in this study seems to possess a cleavage specificity for the α-2-6 glycosidic bond over the α-2-3 glycosidic bond.

A relatively higher content of fucosylated OS was observed in milk from Western Finncattle, Norwegian Doela cattle and Icelandic cattle. These breeds have, in previous studies, been shown to be closely genetically related, supporting the theory that the fucosylated traits may be genetically determined [45]. Levels of free fucose increased with increasing α-fucosidase activity, observed by a positive correlation for α-fucosidase activity and free fucose (r = 0.37, *p* < 0.001), in milk at room temperature. Storage at refrigerated temperatures may thus not be a problem to the breakdown of milk sugars. The correlation is potentially reflecting a release of fucose from other glycans or from fucosylated OS. However, there were no clear correlations between α-fucosidase activity and fucosylated OS to confirm a degradation. The observed correlation of α-fucosidase activity and free fucose was to some degree related to the breeds. This supports the suggestion that mechanisms related to fucosylation are breed-specific, for example, expression of certain fucosyltransferases.

Lactose can serve both as a substrate for β-galactosidases, as well as being released in milk as a product from hydrolysis of larger milk oligosaccharides. The results revealed a relatively weak negative correlation between lactose and β-galactosidase activity, which may reflect a degradation of lactose by milk β-galactosidase. If this is the case, an opposite trend would be expected in the monosaccharides, glucose and galactose. This is however not observed. Glucose and galactose varied more or less along with lactose, which would make sense as monosaccharides are known to increase naturally in milk over lactation in parallel with lactose by lactogenesis [46]. If these free monosaccharides (glucose and galactose) are not the breakdown products from the glycosidases’ actions, they can be leaked from epithelial cells into the mammary lumen during cell apoptosis [47]. Alternatively, they can be degraded by microorganisms in formation of free monosaccharide residues or leaked from the blood due to udder tissue damage either by a mild infection, or due to late lactation allowing metabolites to pass the mammary cell membrane [48,49]. However, due to the high intra-group variation of both lactose, glucose and galactose, these correlations should be interpreted with caution. The reason for these variations in milk sugars is unknown, but may be related to their low abundance in milk in general.

## 4. Materials and Methods

### 4.1. Milk Samples and Sample Collection

Milk samples were collected from individual cows as a part of a Nordic-Baltic Dairy network [10]. A total of 80 samples were collected from eight different native North European breeds with ten individual cows’ milk samples from each breed. The breeds originated from Norway, Denmark, Lithuania, Sweden, Iceland and Finland. The breeds comprised in the study were Norwegian Doela cattle, Norwegian Telemark cattle, Danish Red anno 1970, native Lithuanian Black and White cattle, Swedish Mountain cattle, Icelandic cattle, Eastern Finncattle and Western Finncattle. All breeds were from conventional dairy farms and had mainly been fed with silage and compound feed. Only Norwegian Telemark cattle have been grazing and was milked by hand. The average parity of the cows was three (1–9 calvings) and average lactation stage of 133 days (21–558 days).

### 4.2. Glycosidase Activities

Activities of the following eight glycosidases were measured by 4-methylumbelliferone (4-MU) activity assays: *N*-acetyl-β-glucosaminidase, α-fucosidase, α-galactosidase, β-galactosidase, α-glucosidase, β-glucosidase, *N*-acetyl-α-neuraminidase and β-glucuronidase. The activities were measured in unpasteurized milk using the exact procedure described previously [11]. Analyses were carried out in duplicate. The activity of α-fucosidase was measured at pH 5.0 and the other glycosidases at pH 4.5, using a final substrate concentration of 1 mM. Aliquots of 20 µL milk were mixed with 80 µL fluorogenic substrate and incubated at 37 °C for 1h. Reactions were stopped by 0.25 M glycine-KOH buffer, pH 10.4. Fluorescence of cleaved 4-MU was measured at excitation 400 nm and emission 485 nm using a Multi-mode microplate reader (SynergyTM Mx, BioTek, Winooski, VT, USA) and standard curves were prepared with serially diluted 4-MU (0–50 µM). 

### 4.3. O-Glycan Analysis by Liquid Chromatography-Electrospray Ionization-Mass Spectrometry

*O*-linked glycosylation of GMP present in milk from the native breeds was analyzed using liquid chromatography-electrospray ionization-single-quadrupole mass spectrometry (LC-ESI/MS) after treatment with chymosin. Prior to LC-MS, all samples were skimmed (2000× *g*, 30 min, 4 °C), incubated at 33 °C and treated with chymosin at pH 6.5–6.7 (ChyMax, 200 IMCU/mL, Chr. Hansen, Hørsholm, Denmark) at a final concentration of 0.04 International milk-clotting units (IMCU)/mL for 40 min to cleave ᴋ-CN into para ᴋ-CN and GMP. This was followed by centrifugation (14,000× *g*, 10 min, 4 °C) to isolate the GMP in the supernatant. Of the 80 samples, 10 samples did not coagulate well enough to obtain the GMP-containing supernatant and were thus not analyzed (*n* = 70). The samples were filtered through 0.2 µm filter HPLC vials (Whatman Mini-UniPrep, Sigma Aldrich, St. Louis, MO, USA). The LC-ESI/MS procedure was based on the previously published procedure described by Sunds et al. [42], with the following modifications. A volume of 20 µL was injected into the HPLC system and separated on a BioZen™ Intact XB-C8 column (150 × 2.1 mm, 3.6 µm pore size, Phenomenex, Torrance, CA, USA) operated at 40 °C. The gradient increased from 20% to 34% of Solvent B from 0 to 41 min and with a flow of 0.4 mL min^−1^.

Deconvolution and identification of intact GMP within the mass spectra was conducted using Bioconfirm 10.0 (Mass Hunter Workstation Software, Agilent Technologies, Santa Clara, CA, USA). Due to the expected mass range of 5000–12,000 Da, it was necessary to make use of a hybrid method that uses settings in the intact protein section and in the protein digest section. In the software a standard method was loaded, but with the following changes: for the protein digest method, it was necessary to allow for large masses, thus up to 100 miss-cleavages were allowed and the peptide length range was set to 5–100 amino acids. The MS match tolerance was set to +/− 2.0 Da and *m*/*z* was restricted to 100–3000 *m*/*z*, only peaks with height ≥100 counts were used, and the isotope model was set to peptides. In the intact protein section the charge state was limited to 1–2. For deconvolution the mass range was set to 5000–12000 Da, the mass step was set to 0.05 Da while the *m*/*z* range was limited to 300–3000 *m*/*z* and the MS match tolerance was set to +/− 2.0 Da. The deconvoluted masses were matched against an in-house database of theoretical masses containing GMP of Variants A, B and E with 0–3 phosphorylations and 0–3 glycosylations of the a, b, c, d and e types [26,42]. Figure A1 in Appendix reveals masses, retention times and peak heights identified in milk from Norwegian Doela cattle of individual number 3, which expresses the κ-CN Genotype AA. The figure illustrates an example of the data behind Figure 2, a more comprehensive study on the genetic variation of κ-CN have been described by Jensen et al. [26].

The prevalence of GMP isoforms was based on peak heights from total ion chromatograms, where reported values are means for each breed and each genetic Variants A, B and E. Values are based on the sum of all GMP isoforms identified with a given composition and genetic variant, relative to the total sum of all Variants A, B or E isoforms in each sample, respectively.

### 4.4. Oligosaccharide Extraction and HPLC-Chip/Quadrupole TOF MS

The composition of OS was analysed by HPLC-chip/quadrupole time-of-flight (Q-TOF) MS as described previously by Sunds et al. [10]. Briefly, OS were extracted and purified prior to analysis. Skim milk samples were mixed with four volumes of chloroform:methanol (2:1, *v*/*v*). After centrifugation at 4000× *g* for 30 min at 4 °C, 250 µL of upper methanol solution containing OS was mixed with two volumes of cold pure ethanol. The samples were stored at −30 °C for 1 h to allow for protein precipitation and then centrifuged at 4000× *g* for 30 min at 4 °C. The supernatant containing OS was dried at 30 °C overnight (Genevac MiVac Quattro concentrator, Genevac Ltd., Ipswitch, England), and redissolved in 150 µL 18.2 MΩ-cm (Milli-Q) water (EMD Millipore, Billerica, MA, USA). The OS were further purified by microplate C18 solid-phase extraction (SPE, Glygen Corp., Columbia, MD, USA) to remove peptides. The columns were conditioned with 3 × 100 μL 100% acetonitrile (ACN), followed by 3× 100 μL Milli-Q water. Samples were loaded, and the wells were washed with 600 μL Milli-Q water after sample loading. The eluate containing OS that was collected during and after samples loading was loaded onto a graphitic carbon SPE microplate (Glygen Corp., Columbia, MD, USA) after conditioning the microplate well with 3 × 100 μL 80% ACN containing 0.1% TFA in water, followed by 3 × 100 μL Milli-Q water. The wells were washed with 6 × 200 μL Milli-Q water and the OS were eluted by 3 × 200 μL 40% ACN containing 0.1% TFA in water. The purified OS fraction was dried by centrifugal evaporation and redissolved in 150 µL Milli-Q water. The purified OS isolate was diluted 50 times with Milli-Q water and spiked with 0.5 mg/L xylosyl-cellobiose (Megazyme, Chicago, IL, USA), which served as an internal standard. An Agilent 6520 HPLC-chip/Quadropole TOF MS, equipped with a microfluidic chip cube interface, was utilized for the OS analysis (Agilent Technologies, Santa Clara, CA, USA). The OS separation was achieved using a binary gradient of Solvent A (3% ACN with 0.1% formic acid in water), and Solvent B (89.9% ACN with 0.1% formic acid in water). Aliquots of 2 µL were injected into the LC system with a flow rate of 4 µL/min. The samples were eluted with the following gradient: 0–2.5 min, 0% B; 2.5–20.0 min, 0–16% B; 20.0–30.0 min, 16–44% B; 30.0–35.0 min, 44–100% B; 35.0–45.0 min, 100% B; followed by 0% B for 20 min to equilibrate the column, at a flow rate of 0.3 µL/min. Calibrant ions, with *m/z* 922.009798 and 1221.990637, were applied for in-run calibration. Comprehensive OS libraries were constructed using MS/MS data. Relative abundance of each OS was measured by integration of the area under the curve (AUC) of extracted ion chromatograms using Batch Targeted Feature Extractor from MassHunter Profinder software version B.08.00 (Agilent Technologies, Santa Clara, CA, USA). A mass tolerance of 20 ppm, maximum charge stage of 2, and minimum absolute height of 1000 counts were allowed for the analysis.

### 4.5. Quantification of Monosaccharides and Lactose by High Field Nuclear Magnetic Resonance

Proton ^1^H NMR spectroscopy on a Bruker Avance III NMR operating at 600.13 MHz was used for the analysis of the following sugars: lactose, glucose, galactose, *N*-acetyl-glucosamine, *N*-acetyl-galactosamine, fucose and sialic acid. The method used was previously described by Sundekilde et al. [50]. Skimmed milk samples were filtered using Amicon Ultra centrifugal filter units with a 10 kDa molecular weight cutoff (Millipore, Billerica, MA), at 10,000× *g* for 30 min at 4 °C. The 400 µL permeate was mixed with 200 µL deuterium oxide (D_2_O), containing 0.05% sodium trimethylsilyl-[2,2,3,3-^2^H_4_]-1-propionate (TSP; Sigma-Aldrich A/S, Copenhagen, Denmark), which was employed as an internal chemical shift reference and used for the quantification. NMR spectroscopy was performed as described [50]. Briefly, standard one-dimensional nuclear Overhauser enhancement spectroscopy-presat pulse sequence (noesypr1d) was applied with 64 scans, a spectral width of 7288 Hz, acquisition time of 2.25 s, a total of 32,768 data points, and a relaxation delay of 5 s. The spectra were phase and baseline corrected using Topspin 2.1 (Bruker BioSpin, Rheinstetten, Germany), and the sugars were identified and quantified using Chenomx Profiler NMR Suite 8.3 (Chenomx, Edmonton, AB, Canada). NMR signals used for quantification of monosaccharides are available in Appendix A Table A1 and Table A2.

### 4.6. Data Analysis

Differences in glycosidase activities among the eight native cattle breeds were assessed by a Wilcoxon–Mann–Whitney test, which compares each parameter to the mean of all parameters. Univariate data analysis and visualizations were performed in R version 3.6.1 (R Foundation for Statistical Computing, Vienna, Austria) using the ggplot2, ggpubr and magrittr packages. Multivariate data analysis was performed in SIMCA version 16 (Sartorius Stedim Biotech, Umeå, Sweden) and PLS Toolbox (Eigenvector Research, Inc., Manson, WA, USA). The quantitative data, including glycosidases, monosaccharides, OS, and GMP isoforms, was auto scaled prior to a principal component analysis (PCA). Data on the GMP variants containing the glycosylations (a, b), (b, b), (b, c/d) and (b, b, e) was excluded from the PCA model, due to too few values different from the mean. Further, the GMP isoforms containing similar glycosylations, but with different number of phosphorylations and genotypes were summed to avoid zero values, which would weaken the model.

## 5. Conclusions

Icelandic cattle milk was notable for its relatively high abundance of glycans constituting the functional residues, sialic acid and fucose, and was further low in activities of most glycosidases. On the other hand, breeds representing high glycosidase activities were Norwegian Telemark cattle and native Lithuanian Black and White cattle, which at the same time displayed low abundances of most OS. Several negative correlations between glycosidases and glycan substrates (OS and *O*-linked glycan) were revealed. This could be an indication of degradation of glycans. The strongest relations were observed between *N*-acetyl-β-glucosaminidase activity and the neutral OS, 2 Hex 1 HexNAc, as well as between *N*-acetyl-α-neuraminidase activity and abundance of the acidic OS, 6′-SL. Since no correlation between *N*-acetyl-α-neuraminidase and 3′SL were revealed, the *N*-acetyl-α-neuraminidases measured seemed to be favoring cleavage of α-2-6 glycosidic bonds over α-2-3 glycosidic bonds. Finally, the study indicated a cleavage of fucose by α-fucosidase activities. Hence indigenous milk glycosidases seemed to have an effect on both neutral, acidic and fucosylated glycans, which may have consequences to the recovery of bioactive milk glycans considered as valuable dairy ingredients. The findings of the study may be of inspiration for future breeding strategies favoring certain traits related to valuable glycans, since more knowledge on the milk composition of these endangered breeds is of major importance for an optimal utilization of these unique resources. However, further studies are required to confirm these correlations, preferably in a larger sample set containing fewer intrinsic variables.

## Figures and Tables

**Table 1:** Significant correlations among glycosidases, between glycosidases and glycans, as well as between different specific glycans. Included are Pearson correlation coefficients (r) and corresponding *p*-values.

First Parameter	Correlated Parameter	r	*p*-Value
β-galactosidase activity	β-glucosidase activity	0.79	<0.001
β-glucuronidase activity	β-glucosidase activity	0.73	<0.001
β-glucuronidase activity	β-galactosidase activity	0.61	<0.001
β-glucuronidase activity	*N*-acetyl-β-glucosaminidase activity	0.52	<0.001
*N*-acetyl-β-glucosaminidase activity	2 Hex 1 HexNAc	−0.40	<0.001
*N*-acetyl-α-neuraminidase activity	2 Hex 1 NeuAc (6′-SL)	−0.27	0.024
β-galactosidase activity	Lactose	−0.30	0.007
β-galactosidase activity	GMP (e, e, e)	−0.25	0.030
α-fucosidase activity	Fucose	0.37	<0.001
2 Hex 2 NeuAc	GMP (c/d)	0.36	0.001
3 Hex 1 NeuAc	GMP (c/d)	0.33	0.001

## Data Availability

The data presented in this study are available in article.

## Appendix A

**Table A1:** List of detected glycomacropeptide (GMP) isoforms from Norwegian Doela cattle (individual number 3), to illustrate the isoform composition in a cow of genotype AA. Including retention time (RT), experimental masses, theoretical masses, delta (ppm) and peak heights relative to the total peak heights.

GMP Isoform.	RT	Experimental Mass	Theoretical Mass	Delta (ppm)	Relative Peak Height
A 1P	19.9	6786.6	6787.6	−146.5	0.737
A 1P 1G (b)	18.4	7151.8	7153.0	−158.0	0.003
A 1P 1G (c/d)	17.9	7442.5	7444.2	−224.5	0.005
A 1P 1G (e)	16.8–18.9	7734.0	7735.5	−190.7	0.158
A 1P 2G (c/d, e)	7.1	8390.9	8392.1	−144.8	0.004
A 1P 2G (e, e)	7.6	8682.2	8683.3	−131.0	0.032
A 2P	20.6	6866.5	6867.6	−167.0	0.060

**Table A2:** Assignments of NMR signals used for quantification in Chenomx.

Monosaccaride	δ ppm	Assignment	H
Lactose	3.28	CH-2	1
Glucose	4.63	CH-1	1
Galactose	5.25	CH-1	1
	4.57	CH-1	1
N-acetyl-glucosamine	2.04	CH_3_	3
N-acetyl-galactosamine	2.05	CH_3_	3
Fucose	1.24	CH_3_-6	3
Sialic acid	1.78	CH’-3	1
